# Can Replacing Sitting Time with Standing Time Improve Adolescents’ Cardiometabolic Health?

**DOI:** 10.3390/ijerph16173115

**Published:** 2019-08-27

**Authors:** Bruno P. Moura, Rogério L. Rufino, Ricardo C. Faria, Jeffer E. Sasaki, Paulo Roberto S. Amorim

**Affiliations:** 1 Medical Science Graduate Program, Medical Sciences Faculty, Rio de Janeiro State University, Rio de Janeiro 20550-170, Rio de Janeiro, Brazil; 2 Department of Physical Education, Viçosa Federal University, Viçosa 36570-900, Minas Gerais, Brazil; 3 Graduate Program in Physical Education, Federal University of Triângulo Mineiro, Uberaba 38025-180, Minas Gerais, Brazil

**Keywords:** inclinometer, actigraphy, reallocating time, pediatrics, school health, physical fitness, physical activity, sedentary behavior, public health, metabolic health

## Abstract

This study aimed to assess the effects of isotemporal replacement of sitting time (SIT) with standing (STA) on cardiometabolic biomarkers. In this cross-sectional study, male adolescents wore the GT3X+ activity monitor for 7 days to measure the SIT and STA. Moderate-to-vigorous physical activity (MVPA) was estimated by a youth-specific cut-off point. An isotemporal substitution approach was used to examine the effects of replacing different periods of SIT (15, 30, 60, and 120 min) with STA on cardiometabolic biomarkers [total cholesterol (TC), high-density lipoprotein cholesterol (HDL-c), non-HDL-c, low-density lipoprotein cholesterol (LDL-c), triglycerides (TG), glucose, insulin, HOMA2-β, HOMA2-S, and HOMA2-IR]. Analysis of covariance (ANCOVA) with a post-hoc Bonferroni test was used to compare the adjusted means between the four subgroups that were clustered according to SIT and STA amount. Adolescents (n = 84; age, 16.7 ± 0.9 years) wore GT3X+ for 15.2 ± 2.3 h, for 6.7 ± 0.6 days. Isotemporal substitution of SIT with STA was associated with TC, non-HDL-c, LDL-c, and TG. ANCOVA results showed a statistically significant difference for TC, non-HDL-c, and LDL-c. These findings showed that for male adolescents, sitting less and standing more may be an effective alternative to reduce cardiometabolic biomarker levels related to lipid metabolism, regardless of MVPA.

## 1. Introduction

Adolescents spend more than one-third of their daily waking hours at school [1,2]. Most of this time is spent inside a classroom, where the environment has mostly been developed for sitting activities [3,4,5]. Furthermore, many other daily activities of adolescents, such as school transportation, homework at a desk, eating a meal, playing video games, using a computer, and watching television involve sitting. Recently, accumulating evidence has suggested that sitting time is associated with poor cardiometabolic health outcomes [6,7,8,9,10,11,12], and an increase in all-cause mortality rates and cardiovascular diseases [8,9,13,14], even after adjusting for moderate-to-vigorous physical activity (MVPA) and leisure-time physical activity [7,8,13]. However, these studies were mostly conducted in adults; for children and adolescents, such associations require further evidence [7,8,9,10,11]. Given the ubiquitous nature of sitting in our modern society, recent studies have focused on seeking alternatives to reduce the daily sitting time [2,15,16].

Evidence indicates that both sitting and standing postures provide low levels of energy expenditure but may demand different physiological processes and energy cost [13,15,16]. Standing involves the constant activation of a large muscle mass in the lower limbs and trunk (postural muscles), which become inactive while sitting [15]. Early findings have suggested that standing may be a healthy alternative to reducing sitting time [7,8,15] because the energy expenditure associated with these muscle contractions throughout the whole day can increase the total metabolism [16]. Moreover, unlike sitting time, standing time was associated with improved cardiometabolic health, and a lower risk of all-cause mortality and cardiovascular disease [8,15].

Consequently, broadly established guidelines on sitting time reduction have emerged, and have been discussed for different ages [17,18,19]. For adolescents, these guidelines suggest performing at least 60 min daily of MVPA, as well as spending a maximum of 2 h per day on sedentary behaviors, such as sitting [17,18,19]. Thus, for its practical implementation, it is important to understand the potential benefits of adopting a standing posture, which could replace sitting time. Some recent studies [7,20,21] have used a statistical approach, called isotemporal substitution [22], which allows the study of the effects of replacing sitting time with standing, keeping the total time and time spent in other behaviors fixed. This approach takes into account that time is finite, so the time spent on a given behavior will result in less time spent in another [22]. Evidence acquired through these studies shows that in adults, replacing sitting time with standing was beneficially associated with several cardiometabolic biomarkers, such as plasma glucose, triglycerides, high-density lipoprotein cholesterol (HDL-c), insulin, HOMA-IS, and interleukin-6 [7,20,21]. However, isotemporal replacement has not yet been used to examine the potential impact associated with reallocation of time from sitting to standing, on the cardiometabolic health of adolescents.

Considering that more than 90% of classroom time is spent sitting and that other activities during the waking time of adolescents are also performed while being seated [3,4,5], it becomes imperative to measure the impacts of new alternatives to reduce harmful health outcomes of sitting time. Accordingly, this study aimed to assess the effects of isotemporal replacement of sitting time with standing, on cardiometabolic biomarkers in adolescents.

## 2. Materials and Methods

### 2.1. Design and Participants

This cross-sectional study was conducted in Brazil in 2013, through a convenience sample composed of male adolescents enrolled at the Federal Institute of Education, Science and Technology, Rio Pomba Campus. In 2013, the Institute had 424 students enrolled (59.2% male). As the Institute only provided student housing for male students, there were only 140 residents in the student housing. We chose this population because of a greater standardization of habits, as all students had to follow Institute-standardized times for waking up, studying, eating, sports, leisure, and sleeping. After a meeting with these adolescents to introduce the research project, only those with the following characteristics were eligible to participate: (i) age between 14 and 18 years; and (ii) provided written informed consent for participation in this study (informed consent for those aged <18 years was signed by parents or guardians). The exclusion criteria were as follows: (i) use of medication for cardiometabolic conditions; (ii) previously diagnosed metabolic diseases; (iii) reports of severe cardiovascular disease or other comorbidities leading to functional disability; or (iv) calorie-restricted diet. This research was conducted in accordance with the Declaration of Helsinki, and the protocol was approved by the Ethics Committee on Human Research of the Federal University of Viçosa (N°. 0100/2012).

### 2.2. Physical Activity and Body Position Measures

The GT3X+ activity monitor (ActiGraph Corp, Pensacola, FL, USA) was used to measure the physical activity and determine the body position information. This is a small device with an accelerometer based on the microelectromechanical system, able to measure triaxial acceleration (*x*, *y*, and *z* axes) within a range of ± 6 Gs at a sampling rate of 30 to 100 Hz, and provides objective measurements of human activity with high reliability [23].

Adolescents were instructed to wear the GT3X+ on the right side of the waist (aligned with the axillary line of iliac crest) fixed by an elastic belt for 7 consecutive days during daily waking hours and to remove it only to sleep at night or during water-based activities (e.g., bathing or swimming) [24]. Written instructions on the correct use of the device and the researcher’s contact information were provided to everyone. Furthermore, all participants were advised not to change their daily routine. The activity data were collected at a 30 Hz sampling rate and post-processed using the ActiLife software (v6.13.3) (ActiGraph Corp, Pensacola, FL, USA). All files were converted to a 15 s epoch length without the use of the low-frequency extension filter [24]. Non-wear time was assessed by an automated algorithm, considering a minimum length of 60 min, a small window length of 30 min, and spike tolerance of 2 min [25]. A valid day was defined as wear time of ≥480 min·day^−1^ (8 h·day^−1^), and only data with at least three valid days (at least 2 weekdays and 1 weekend day) were included for further analysis [26]. The “Sleep period” and “Ignore first sedentary Break of each day” options contained in the ActiLife 6 software were selected and therefore such periods were marked as non-wear time and excluded from further analysis. Accelerometer data from the 3-axis were combined into a vector magnitude (VM), and MVPA time (min·day^−1^) was estimated by a VM activity count, with cut-off point specifically validated for Brazilian adolescents (≥3028 counts·min^-1^) [24]. Based on continuous MVPA values, participants were categorized as either “met MVPA guidelines (≥60 min·day^−1^)” or “did not meet MVPA guidelines” [17,18,19]. Sitting, lying, and standing time of each adolescent during daily waking hours was provided by the inclinometer functionality of the GT3X+ device, which determined the posture by calculating two angles obtained from two algorithms, taking into account the acceleration on each 3-axis of motion (*x*, *y*, and *z*) [27,28,29].

### 2.3. Anthropometric, Demographic, and Blood Pressure Measures

Information about age, ethnicity, and current smoking status was obtained by an interview-based questionnaire. Age was determined as a continuous variable from birth to the intervention date. Ethnicity was coded as white Latin Americans or non-white Latin Americans, and the current smoking status (if they smoked any type of cigarette in the last 3 months) was coded as smokers or non-smokers. Measurements of body weight (kg), height (m), waist circumference (cm), and triceps skinfold (mm) were obtained by a trained technician, according to Lohman and colleagues [30]. Body mass index (BMI) was coded as normal or altered weight (overweight and/or obesity) according to standard guidelines [31]. Waist circumference (WC) was assessed at the midpoint between the last rib and the iliac crest, as recommended by previous studies [30]. Fat mass (FM) was predicted by an adolescent-specific equation using triceps skinfold [32], which was measured in duplicate with the Lange Skinfold Caliper (Beta Technology, Santa Cruz, CA, USA), and was shown as percentage of body weight [FM (% weight)]. In addition to anthropometry, systolic- (SBP) and diastolic-blood pressure (DBP) were measured by a trained technician, according to standard guidelines [33]. Adolescents who presented SBP and/or DBP above the 95th percentile, according to age, sex, and height percentile were classified as having high-blood pressure [33].

### 2.4. Cardiometabolic Biomarkers Measurement

Blood samples (5 mL) were collected after a fasting period of 12–14 h (between 6:00 and 7:00) from the median cubital vein by trained professionals. Serum glucose, total cholesterol (TC), high-density lipoprotein cholesterol (HDL-c), and triglycerides (TG) were measured by the enzymatic colorimetric method. Insulin level was measured using the electrochemiluminescence method. Analyses were performed with the biochemical analyzer ChemWell^®^-T (Awareness Technology^®^, Palm City, FL, USA). Low-density lipoprotein cholesterol (LDL-c) and non-HDL-c were determined according to previous studies [34,35]. Insulin resistance (HOMA2-IR), insulin sensitivity (HOMA2-S), and beta cell function (HOMA2-β) were assessed by the Homeostasis Model Assessment (HOMA2) and were calculated by the HOMA2 calculator (version 2.2.3, University of Oxford, Oxford, UK) [36].

### 2.5. Statistical Analysis

All statistical analyses were performed using IBM SPSS Statistics 24 (IBM Corporation, Armonk, NY, USA) with statistical significance of *p* < 0.05. Data normality was checked by the Shapiro–Wilk test and owing to skewed distributions, log transformations were performed on HDL-c, TG, insulin, HOMA2-β, HOMA2-S, and HOMA2-IR. Descriptive statistics were used to summarize the characteristics of adolescents, with data presented as mean and standard deviation (SD) for variables with normal distribution and as median and interquartile range (IQR) for ones with non-normal distribution.

Force-entry multivariate linear regression modeling employing an isotemporal substitution [22] approach was used to verify the associations of substituting the same amount of sitting time with standing on cardiometabolic biomarkers. This approach was applied on time blocks of 15, 30, 60, and 120 min. The choice of these time blocks, which are multiples of the quarter of an hour, was due to the fact that normally in the school environment the activities (lessons) last approximately 1 h. The statistical power for multivariate linear regression models (two-tailed; effect size = 0.2; α-error = 0.05) and for analysis of covariance (effect size = 0.3; α-error = 0.05) was based on a post-hoc analysis performed on G*Power (v.3.1.9.2).

Additionally, the continuous variables, sitting and standing times, were labeled as “Low” and “High”, according to their means. Participants with a mean sitting time greater than or equal to 391.8 min·day^−1^ were considered Sitting-High and those who had a mean standing time greater than or equal to 409.2 min·day^−1^ were considered Standing-High. Subsequently, adolescents were clustered based on the amount of sitting and standing time into four distinct subgroups: (i) Sitting-High–Standing-Low; (ii) Sitting-High–Standing-High; (iii) Sitting-Low–Standing-Low; (iv) Sitting-Low–Standing-High. Analysis of covariance (ANCOVA) with a post-hoc Bonferroni test was used to compare the adjusted means between these four subgroups for those biomarkers that presented statistical significance in the previously applied multivariate linear regression analysis.

In both statistical tests, all associations were adjusted for daily awake hours, device wear (days), age, smoking status, BMI, and MVPA daily recommendation (met or not met MVPA guidelines). In addition, all assumptions required for multivariate linear regression and ANCOVA were verified, including linearity and multicollinearity.

## 3. Results

### 3.1. Descriptive Characteristics

A total of 109 adolescents were initially considered for the study, and 92 agreed to participate. Eight participants were excluded from further analysis due to missing data of the GT3X+ device on at least one weekend day. Thus, the final sample size of this study was composed of 84 Brazilian adolescents, which provided a relevant post-hoc statistical power (0.98 for multivariate linear regression models and 0.77 for ANCOVA). The descriptive characteristics of this sample are presented in Table 1. Eighty-three percent of adolescents were white Latin Americans, and 95.2% reported as non-smokers. Overall, they were in good health, mean values of biomarkers were within the normal range (except for TG, classified as borderline), and approximately 94% were of normal weight and normotensive.

On average, the GT3X+ monitor was worn for 15.2 ± 2.3 h of daily waking hours, as shown in Table 1, on 6.7 ± 0.6 days, ranging from 4 to 7 days (1.2% wore for 4 days, 5.9% for 5 days, 15.5% for 6 days, and 77.4% for 7 days). Therefore, 92.9% of these data comes from 6 or 7 valid days. Furthermore, the GT3X+ triaxial data analysis revealed that 90.5% of these adolescents met the daily MVPA recommendations.

### 3.2. Replacing Sitting Time with Standing Time

**Figure 1:**
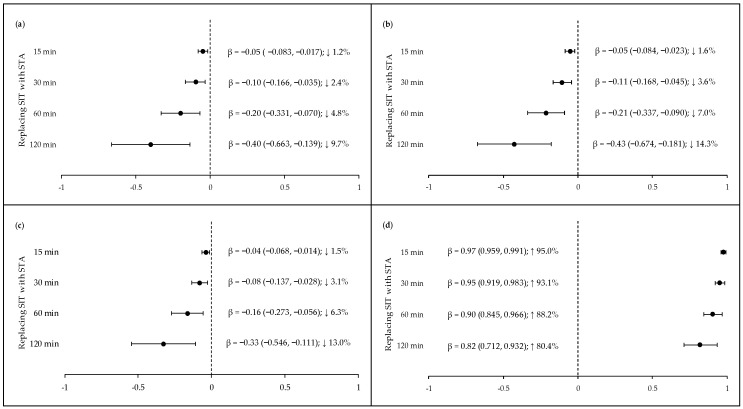
Effects of the isotemporal substitution of sitting time with standing time on cardiometabolic biomarkers. (**a**) Total cholesterol (TC); (**b**) non-HDL-cholesterol (Non-HDL); (**c**) low-density lipoprotein cholesterol (LDL-c), and (**d**) triglycerides (TG). Note: SIT: sitting time; STA: standing time; β (lower 95% CI, upper 95% CI); CI: confidence interval; ↓: % decrease in variable relative to the mean value; ↑: % increase in variable relative to the mean value. All models were adjusted for daily waking hours, device wear (days), age, smoking status, body mass index (BMI), and moderate-to-vigorous physical activity (MVPA) daily recommendation. Data of TG were transformed from the log scale for better interpretation. All bouts had a *p*-value < 0.05 and the dashed line indicates no effect. Power (1−β err prob) = 0.98.

**Figure 2:**
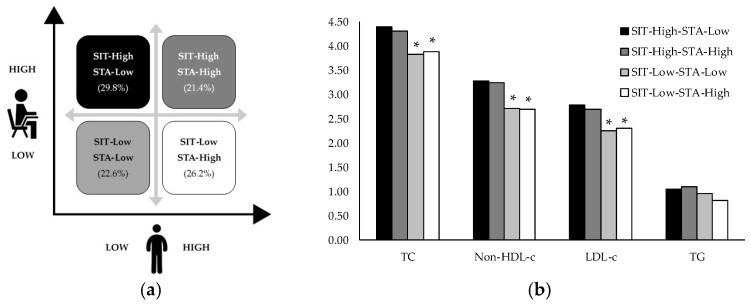
Clusters of sitting time with standing (**a**) and cardiometabolic health outcomes of adolescents within each cluster (**b**). Note: TC: total cholesterol; Non-HDL-c: non-HDL-cholesterol; LDL-c: low-density lipoprotein cholesterol; TG: triglycerides; SIT: sitting time; STA: standing time. * *p* < 0.05 vs. SIT-High; STA-Low. Power (1−β err prob) = 0.77. All models were adjusted for daily waking hours, device wear (days), age, smoking status, body mass index (BMI), and MVPA daily recommendation. Data of TG were transformed from the log scale for better interpretation.

The isotemporal substitution of sitting time with standing was associated with TC, Non-HDL-c, LDL-c, and TG, as shown in Figure 1. Such associations were evident in time blocks of 15 min and evolved linearly until the time blocks of 120 min. The replacement of sitting time with standing showed a decrease in serum levels of TC, non-HDL-c, and LDL-c; however, for TG, this change caused an increase. No statistical significance was found for HDL-c, glucose, insulin, HOMA2-β, HOMA2-S, and HOMA2-IR. When adolescents were clustered based on the daily amount of time spent sitting and standing, as shown in Figure 2a, 29.8% were coded as Sitting-High–Standing-Low, 21.4% as Sitting-High–Standing-High, 22.6% as Sitting-Low–Standing-Low, and 26.2% as Sitting-Low–Standing-High. When compared with the individuals that were allocated in the Sitting-High–Standing-Low group, those coded as Sitting-Low–Standing-High had a reduction of 11.6%, 18.0%, 17.0%, and 22.2% in the mean levels for TC, non-HDL-c, LDL-c, and TG, respectively. However, the ANCOVA results identified a statistical difference for TC, non-HDL-c, and LDL-c. TG was the only variable with no statistical significance, as shown in Figure 2b. The datasets generated and/or analyzed during the current study are available on Mendeley Data repository (http://dx.doi.org/10.17632/svv8f82rn5.2) [37].

## 4. Discussion

To our knowledge, this is the first study to examine the possible effects of isotemporal replacement of sitting time with standing on cardiometabolic biomarkers in adolescents. Our results suggested that standing time was beneficially associated with cardiometabolic biomarkers related to lipid metabolism, but not with those of carbohydrate metabolism. Such benefits were evidenced by the replacement of only 15 min of sitting time, and its magnitude increased linearly up to 120 min. Thus, sitting less and standing more may be one of the measures to reduce serum levels of TC, non-HDL-c, and LDL-c regardless of MVPA. In the TG analysis, it was observed that the replacement of sitting time with standing led to an increase in serum TG level. This result was somewhat unexpected, since the presumed hypothesis was that the enzymatic actions triggered by the muscular contractions necessary to assume the standing posture would cause a reduction of TG levels [9,38]. Therefore, it should be noted that the TG baseline was labeled as borderline, and this may have influenced this outcome. However, on analyzing its evolution over all time blocks (from 15 min to 120 min), a reduction in the level was observed.

This study has extended the results of previous research [7,20,21] conducted in adults and the elderly, to adolescents. Such studies have shown that isotemporal replacement of sitting time with standing may provide improvements in cardiometabolic health [7,20,21]. Edwardson et al. [20] identified that the replacement of 30 min of sitting time with standing was associated with a 4% reduction in fasting insulin, and a 4% increase in HOMA-IS, and a 5% increase in the Matsuda-Insulin Sensitivity Index. Henson et al. [21] found a 4% reduction in interleukin-6 levels on replacing 60 min of sitting time with standing, while Healy et al. [7] showed that the replacement of 120 min was associated with significantly lower fasting glucose (2%), total/HDL-c ratio (6%), TG (11%), and higher HDL-c (0.06 mmol/L). Although the evidence is limited [7,20,21], replacing different periods of sitting time with standing appear to have positive impacts on metabolic health of adults, older adults, and now for male adolescents. Our findings indicated that replacement of 30, 60, or 120 min of sitting time with standing promotes a reduction of 2.4%, 4.8%, and 9.7% for TC; 3.6%, 7.0%, and 14.3% for non-HDL-c; and 3.1%, 6.3%, and 16.2% for LDL-c. These findings are consistent with previous epidemiological and experimental studies [7,9,20,21,39,40,41], which show that reducing daily sitting time by approximately 60 min may probably be the minimum necessary to obtain clinical benefits, with further reductions resulting in greater health gain. Therefore, along with messages related to the accumulation of at least 60 min·day^−1^ of MVPA, adolescents should also be encouraged to sit less and stand more.

In this context, standing time may be considered an effective alternative to reduce the amount of time spent sitting daily, including the time spent in school [2,15]. These findings corroborate evidence from previous studies that observed a decrease in sitting time among adolescents upon the use of standing desks in the classroom setting, without undermining the cognitive process [2,3]. Based on the evidence from our study, if 15 min of each study session (usually five in the morning) were held in a standing posture, this could promote a 75 min reduction in sitting time, and consequently, a probable improvement in the cardiometabolic health of these students. Often, teachers, principals, and education departments have cited the existence of a crowded curriculum as the reason for not implementing physical activity programs in schools [2]. Taken together, these results suggest that small structural changes within the classrooms (e.g., the use of standing desks) may provide a great opportunity for young people to reduce sitting time and improve health outcomes [2,3].

However, the present study shows a divergence from the studies conducted by Edwardson et al. [20] and Healy et al. [7]. Our findings identified improvements only in biomarkers linked with lipid metabolism but not with carbohydrate metabolism. Other studies have also shown an improvement in biomarkers associated with carbohydrate metabolism [7,20]. A possible explanation for this divergence is that the samples used in the two aforementioned studies [7,20] were composed of people at high risk for type 2 diabetes and therefore had high baseline levels of carbohydrate-related biomarkers. Other potential physiological mechanisms that may explain the standing time benefits on biomarkers linked to lipid metabolism exist [9,15,38]. Studies have suggested that increased activity of lipoprotein lipase and hormone-sensitive lipase, enzymes responsible for the hydrolysis of triglyceride-rich lipoproteins during muscle contractions, promote the breakdown of TGs into free fatty acids, reducing TGs in circulation [9,38]. This fact may also explain the decrease observed for TG when the replacement time was elevated from 15 min to up to 120 min. Free fatty acids are the main fuel for slow-twitch muscle fibers (type I), which have high oxidative and low glycolytic capacities. These muscle fibers are relatively resistant to fatigue and are predominantly recruited by the postural muscles during the standing position [9,15]. Moreover, standing may also disrupt the reductions in shear stress in the lower limbs occurring during sitting, and potently improve the endothelial function [15].

The objective measurement of adolescents’ sitting and standing time and its relationship with health outcomes can be considered one of the main strengths of this study. However, there are some limitations that should be mentioned. As seen in other cross-sectional research, the causative factors for the observed results cannot be determined. Although this study has adequate statistical power for both tests, its relatively small sample size may have affected the magnitude of the results and the detection of significant associations for some outcomes. The TG baseline, which was classified as borderline, may have impacted the associations of this variable. The isotemporal substitution model itself may also be considered a limitation because like any mathematical model, its results may not reflect the real world. Moreover, the fact that the sample was composed only of male adolescents did not allow any inference to female adolescents. Finally, the algorithm used to determine the GT3X+ post-processed positional detection appears to be more accurate for thigh-worn devices than waist-worn [28], and this may have affected the outcomes. However, considering the large numbers of studies and databases generated by waist-worn devices around the world, this opens a window of opportunity for new analyses.

## 5. Conclusions

This study provides new evidence for the potential cardiometabolic health benefits of male adolescents by replacing sitting time with standing time. These findings showed that sitting less and standing more may be an effective alternative to reduce the cardiometabolic biomarker levels related to lipid metabolism, regardless of MVPA. Furthermore, these results corroborate previous studies that advocate for the use of standing desks within the school classroom as a way to reduce daily sitting time. However, further studies addressing the isotemporal substitution model, as well as other models of analysis (e.g., compositional analysis) [42], are needed to understand the issues related to behavioral co-dependency within a finite period of time. Therefore, studies that include 24 h data collection protocols in female and male adolescents with different experimental designs are needed.

## Figures and Tables

**Table 1:** Descriptive characteristics of adolescents.

Variables (n = 84)	Mean	(SD)
Age (years)	16.69	(0.93)
Weight (kg)	62.62	(9.56)
Height (m)	1.74	(0.06)
BMI (kg·m^−2^)	20.59	(2.87)
WC (cm)	73.51	(6.59)
FM (% weight)	24.33	(3.73)
TC (mmol/L)	4.11	(0.62)
HDL-c (mmol/L) *	1.11	(0.28)
Non-HDL-c (mmol/L)	2.99	(0.58)
LDL-c (mmol/L)	2.52	(0.51)
TG (mmol/L) *	0.95	(0.41)
Glucose (mmol/L)	4.32	(0.42)
Insulin (pmol/L) *	35.18	(43.53)
HOMA2-β (%) *	102.35	(64.80)
HOMA2-S (%) *	156.65	(141.50)
HOMA2-IR *	0.64	(0.82)
SBP (mmHg)	111.35	(11.21)
DBP (mmHg)	72.38	(7.81)
Daily waking hours (DWH)	15.21	(2.32)
Device wear (days)	6.69	(0.64)
Sitting time (min·day^−1^)	391.79	(81.43)
Lying time (min·day^−1^)	111.32	(75.73)
Standing time (min·day^−1^)	409.20	(89.98)
Sitting time (% of DWH)	43.16	(7.71)
Lying time (% of DWH)	11.68	(6.57)
Standing time (% of DWH)	45.16	(9.16)

Note: BMI: body mass index; WC: waist circumference; FM: fat mass; TC: total cholesterol; HDL-c: high-density lipoprotein cholesterol; Non-HDL-c: non-high-density lipoprotein cholesterol; LDL-c: low-density lipoprotein cholesterol; TG: triglycerides; HOMA2-β: homeostatic model assessment—beta cell function; HOMA2-S: homeostatic model assessment—insulin sensitivity; HOMA2-IR: homeostatic model assessment—insulin resistance; SBP: systolic-blood pressure; DBP: diastolic-blood pressure; SD: standard deviation; and min: minutes. * Data are presented as median and interquartile range (IQR).
